# Molecular Characterization of Hypothalamic–Pituitary–Ovarian Axis Regulation in the Manchurian Zokor (*Myospalax psilurus*) During Seasonal Estrus

**DOI:** 10.3390/genes16111289

**Published:** 2025-10-30

**Authors:** Rile Nai, Xueru Li, Dan Shan, Saru Bao, Fei Wang, Yuerong Lin, Yan Zhang, Buqin Hu, Yuchun Xie, Duhu Man

**Affiliations:** 1 College of Agriculture, Hulunbuir University, Hulunbuir 021008, China; nairile514@126.com (R.N.);; 2 Hebei Key Laboratory of Specialty Animal Germplasm Resources Exploration and Innovation, College of Animal Science and Technology, Hebei Normal University of Science and Technology, Qinhuangdao 066004, China; 3 Hulunbuir Academy of Inland Lakes in Northern Cold & Arid Areas, Hulunbuir 021008, China

**Keywords:** Manchurian zokor, seasonal reproduction, transcriptome, HPO axis, estrus, anestrus

## Abstract

Background/Objectives: Seasonal reproduction in mammals is primarily regulated by the hypothalamic–pituitary–ovarian (HPO) axis, yet its molecular mechanisms in subterranean rodents living in light-restricted environments remain poorly understood. This study aimed to characterize the transcriptional regulation of the HPO axis during seasonal estrus in the Manchurian zokor (*Myospalax psilurus*, *M. psilurus*), a fossorial rodent exhibiting distinct breeding cycles despite perpetual darkness. Methods: Hypothalamic, pituitary, and ovarian tissues were collected from female zokors during estrus and anestrus (*n* = 5 per group). RNA sequencing was performed, followed by de novo transcriptome assembly and bioinformatic analyses. Differentially expressed genes (DEGs) were identified using edgeR, and functional enrichment was assessed via GO and KEGG analyses. Key DEGs were validated by RT-qPCR. Results: A total of 513, 292, and 138 DEGs were identified in the hypothalamus, pituitary, and ovary, respectively. GO analysis highlighted enrichment in G-protein-coupled receptor signaling, oxidation–reduction processes, and calcium ion binding. KEGG pathway analysis revealed significant enrichment of the neuroactive ligand–receptor interaction pathway across all three tissues. Key candidate genes included *Trh* and *Mc3r* in the hypothalamus, *Pitx2* and *NR4A2* in the pituitary, and *PTGER2* and *Sphk1* in the ovary. Conclusions: This study provides the first comprehensive transcriptomic profile of the HPO axis in Manchurian zokors during seasonal estrus. The neuroactive ligand–receptor interaction pathway appears central to reproductive regulation, and several tissue-specific genes were identified as potential regulators of seasonal breeding. These findings enhance our understanding of reproductive adaptation in subterranean mammals and offer a foundation for further functional studies.

## 1. Introduction

Reproduction is essential for species survival and population persistence, with seasonal reproduction being a common strategy in mammals. This process is primarily regulated by the hypothalamus–pituitary–ovarian (HPO) axis and entrained by environmental cues—such as light–dark cycles—via the suprachiasmatic nucleus [1,2,3]. The HPO axis modulates follicular development, ovulation, and estrogen synthesis through coordinated feedback mechanisms involving gonadotropin-releasing hormone (GnRH), luteinizing hormone (LH), and follicle-stimulating hormone (FSH), while reproduction-related genes exhibit rhythmic expression that supports seasonal breeding [4,5,6,7,8,9,10].

Subterranean rodents provide a unique model for studying seasonal reproduction, as their underground habitats differ drastically from the aboveground environments in which most mammalian reproductive rhythms have been studied [11]. The Manchurian zokor, a typical subterranean rodent distributed across Transbaikalia, eastern Mongolia, and eastern and central China, resides in underground tunnels year-round yet still exhibits a distinct seasonal breeding pattern—breeding initiates in late spring and pup rearing occurs in summer [12,13,14]. However, how seasonal reproduction is regulated under lightless conditions remains unclear. The molecular mechanisms underlying this process, particularly the transcriptional dynamics across the HPO axis, are still unknown.

In the present investigation, hypothalamic, pituitary, and ovarian tissue samples were collected from female Manchurian zokors during estrus and anestrus, from which mRNA was extracted and subjected to separate sequencing. Bioinformatic analysis of the resulting data identified key genes and signaling pathways that regulate seasonal reproduction throughout the entire HPO axis of the Manchurian zokor.

## 2. Materials and Methods

### 2.1. Experimental Animals and Tissue Collection

Manchurian zokors were captured in the Hulunbeier Meadow Grassland Area in Inner Mongolia, China (E 120°20′1″–120°16′1″, N 49°14′–49°20′), using circular-tong traps during the non-breeding (mid-April 2024) and breeding (May 2024) seasons. From the captured individuals, five females in the breeding stage and five in the non-breeding stage, with a mean body weight of 335.1 ± 21.4 g, were selected for subsequent analyses. The sample size for each group (*n* = 5) followed standard practice in similar mammalian transcriptomic studies, providing a balance between statistical robustness for detecting differentially expressed genes and the ethical principle of minimizing animal use [5,15]. Female zokors captured in each season were randomly assigned to experimental groups. The random allocation sequence was generated using the random-number function in Microsoft Excel. To minimize potential confounding factors, the order of tissue dissection, RNA extraction, and library preparation for all samples was also randomized. Sample positions during sequencing were likewise randomized to avoid batch effects. Animals were euthanized by carbon dioxide inhalation. Hypothalamic, pituitary, and ovarian tissues were rapidly dissected, flash-frozen in liquid nitrogen, and stored at −80 °C until RNA isolation.

Group allocation was necessarily known during tissue dissection; however, to prevent bias, all subsequent laboratory procedures—including RNA extraction, library construction, RNA sequencing, and qPCR—and all data analyses were performed under blinded conditions. Samples were handled using coded labels by personnel unaware of group identity until data collection and analyses were complete.

### 2.2. RNA Extraction and Library Construction

Total RNA was extracted from hypothalamic, pituitary, and ovarian tissues of *Manchurian zokors* using TRIzol reagent (Invitrogen, Carlsbad, CA, USA). The extraction procedure included tissue homogenization in TRIzol, phase separation with chloroform, RNA precipitation with isopropanol, and washing with 75% ethanol. The RNA pellet was air-dried and dissolved in RNase-free water. RNA concentration and purity were determined with a NanoDrop ND-1000 spectrophotometer (NanoDrop, Technologies, Wilmington, DE, USA), and integrity was verified using an Agilent Bioanalyzer 2100 (RIN > 7.0, Agilent Technologies, Santa Clara, CA, USA) and denaturing agarose gel electrophoresis. Polyadenylated RNA was purified from 1 μg of total RNA using Dynabeads™ Oligo(dT)25 (Thermo Fisher Scientific, Waltham, MA, USA) in two rounds. The enriched poly(A) RNA was fragmented at 94 °C for 5–7 min using a Magnesium RNA Fragmentation Module (New England Biolabs, Ipswich, MA, USA). First-strand cDNA was synthesized with SuperScript™ II Reverse Transcriptase (Invitrogen, Carlsbad, CA, USA), and the second strand was generated using *Escherichia coli* (*E. coli*) DNA Polymerase I (NEB, Ipswich, MA, USA), RNase H (NEB, USA), and dUTP Solution (Thermo Fisher, USA) to incorporate dUTP. The resulting cDNA fragments were blunt-ended, adenylated at their 3′ ends for adapter ligation, and size-selected using AMPureXP beads. Uracil bases were excised using UDG enzyme (NEB, USA), and the libraries were PCR-amplified (95 °C for 3 min; 8 cycles of 98 °C for 15 s, 60 °C for 15 s, and 72 °C for 30 s; final extension at 72 °C for 5 min). The final cDNA libraries, with an average insert size of 300 ± 50 bp, were sequenced (paired-end, 2 × 150 bp) on an Illumina NovaSeq™ 6000 platform at LC Bio (Hangzhou, China).

### 2.3. Identification of Differentially Expressed Genes

All biological replicates (*n* = 5 per group) were processed and analyzed independently throughout RNA extraction, library preparation, sequencing, and differential expression analysis to ensure statistical robustness and reproducibility. Following quality control, raw sequencing reads were processed with the fastp tool using default parameters to remove adapter contaminants, low-quality bases, and undetermined nucleotides. In the absence of a reference genome for Manchurian zokor, the clean reads were subjected to de novo assembly using Trinity (Version 2.15). The assembled unigenes were functionally annotated by aligning them against six public databases—Nr, GO, Swiss-Prot, KEGG, Pfam, and eggNOG—using DIAMOND with a stringent *E*-value cutoff of <0.00001. For expression quantification, sample-specific transcriptomes were first assembled with StringTie and then merged into a unified transcriptome using gffcompare. Transcript abundance was estimated as FPKM values via StringTie, and differential expression analysis was performed using the edgeR package (Version 3.40.2). Differentially expressed genes (DEGs) were identified using a false discovery rate (FDR) of 1% for statistical reliability, with additional thresholds of an absolute fold change > 1.5 and a p-value < 0.05.

### 2.4. GO and KEGG Enrichment Analysis

Functional enrichment of DEGs was investigated through Gene Ontology (GO) and Kyoto Encyclopedia of Genes and Genomes (KEGG) analyses using the DAVID database and the ClueGO (Version 3.10.2) plugin in Cytoscape (Version 3.10.2). Terms with a corrected *p*-value < 0.05 were considered statistically significant. GO enrichment results were visualized as bar graphs showing distributions across biological processes (BP), cellular components (CC), and molecular functions (MF).

### 2.5. Validation of DEGs by RT-qPCR

To validate the RNA-seq results, nine DEGs were randomly selected for confirmation by quantitative real-time PCR (qRT-PCR). Following cDNA synthesis from total RNA using the PrimeScript RT Reagent Kit (TaKaRa, Kusatsu, Japan), qRT-PCR assays were performed with SYBR Premix Ex Taq (TaKaRa, Japan) and gene-specific primers Gene expression was normalized to *GAPDH* (Supplementary Materials S1) as an internal control, and relative expression levels were calculated using the 2^−ΔΔCt^ method. Data are presented as the mean ± SEM from five biological replicates, with each sample run in triplicate.

### 2.6. Statistical Analysis

All statistical analyses were performed using SPSS software (version 23.0; IBM Corp., Armonk, NY, USA). Continuous variables are presented as the mean ± SEM. Between-group differences were determined using Student’s *t*-test, with * *p* < 0.05 and ** *p* < 0.01 indicating two levels of statistical significance.

## 3. Results

### 3.1. RNA Sequencing Data Quality

**Figure 1:**
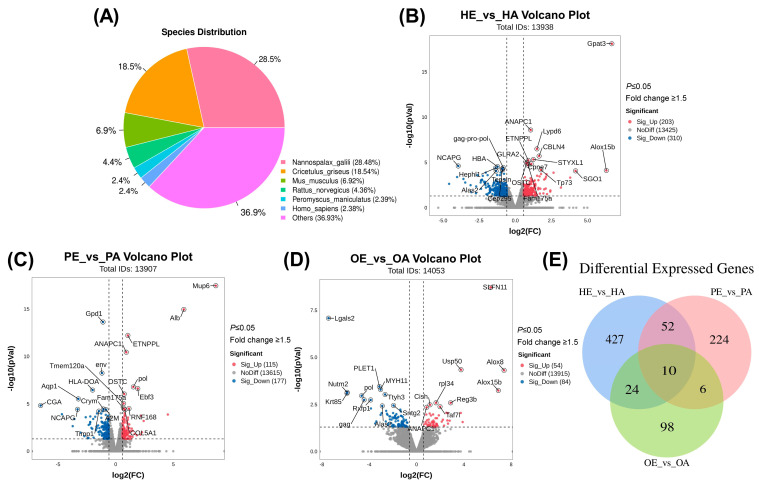
Transcriptomic profiling of hypothalamic–pituitary–ovarian-axis tissues in the Manchurian zokor during estrus and anestrus. (**A**) Species distribution from BLASTX homology analysis of assembled unigenes. Nannospalax galili shows the highest homology, followed by Cricetulus griseus, Mus musculus, Rattus norvegicus, and Peromyscus maniculatus. (**B**) Volcano plot of differentially expressed genes in the hypothalamus (HE_vs_HA). (**C**) Volcano plot of differentially expressed genes in the pituitary (PE_vs_PA). (**D**) Volcano plot of differentially expressed genes in the ovary (OE_vs_OA). *X*-axis: log2 (fold change), *y*-axis: −log10 (*p*-value). Tags in red represent upregulated genes during estrus, and tags in green represent downregulated genes during estrus. (**E**) Venn diagram illustrating the overlap of differentially expressed genes among hypothalamus, pituitary, and ovarian tissues.

Transcriptomic analysis was performed on hypothalamic, pituitary, and ovarian tissues from Manchurian zokors during estrus and anestrus. A total of 15 cDNA libraries representing all groups were constructed and sequenced on the Illumina NovaSeq™ 6000 platform. After quality control, each library yielded an average of over 41 million raw reads and 40 million clean reads, with overall data quality meeting established standards (Table 1). Based on (Version 2.16.0) similarity analysis, approximately 60.1% of the assembled unigenes were functionally annotated to known rodent genes. The annotated sequences showed the highest homology with those of the blind mole-rat (*Nannospalax galili*), Chinese hamster (*Cricetulus griseus*), mouse (*Mus musculus*), rat (*Rattus norvegicus*), and deer mouse (*Peromyscus maniculatus*) (Figure 1A).

### 3.2. Differentially Expressed Gene Identification

To explore the molecular mechanisms regulating the HPO axis during the estrous cycle in Manchurian zokors, we compared gene expression profiles across three tissue-specific comparisons: HE vs. HA (hypothalamus), PE vs. PA (pituitary), and OE vs. OA (ovary). In the hypothalamus, 513 mRNAs were differentially expressed between estrus and anestrus, with 203 upregulated and 310 downregulated genes (Figure 1B, Supplementary Materials S2). The pituitary exhibited 292 DEGs (115 upregulated, 177 downregulated; Figure 1C, Supplementary Materials S3), while the ovary showed 138 DEGs (54 upregulated, 84 downregulated; Figure 1D, Supplementary Materials S4). Venn diagram analysis identified 10 DEGs common to all three tissues, with tissue-specific DEG counts of 427, 224, and 98 in the hypothalamus, pituitary, and ovary, respectively (Figure 1E).

### 3.3. GO Enrichment Analysis of DEGs in HPO-Axis Tissues

**Figure 2:**
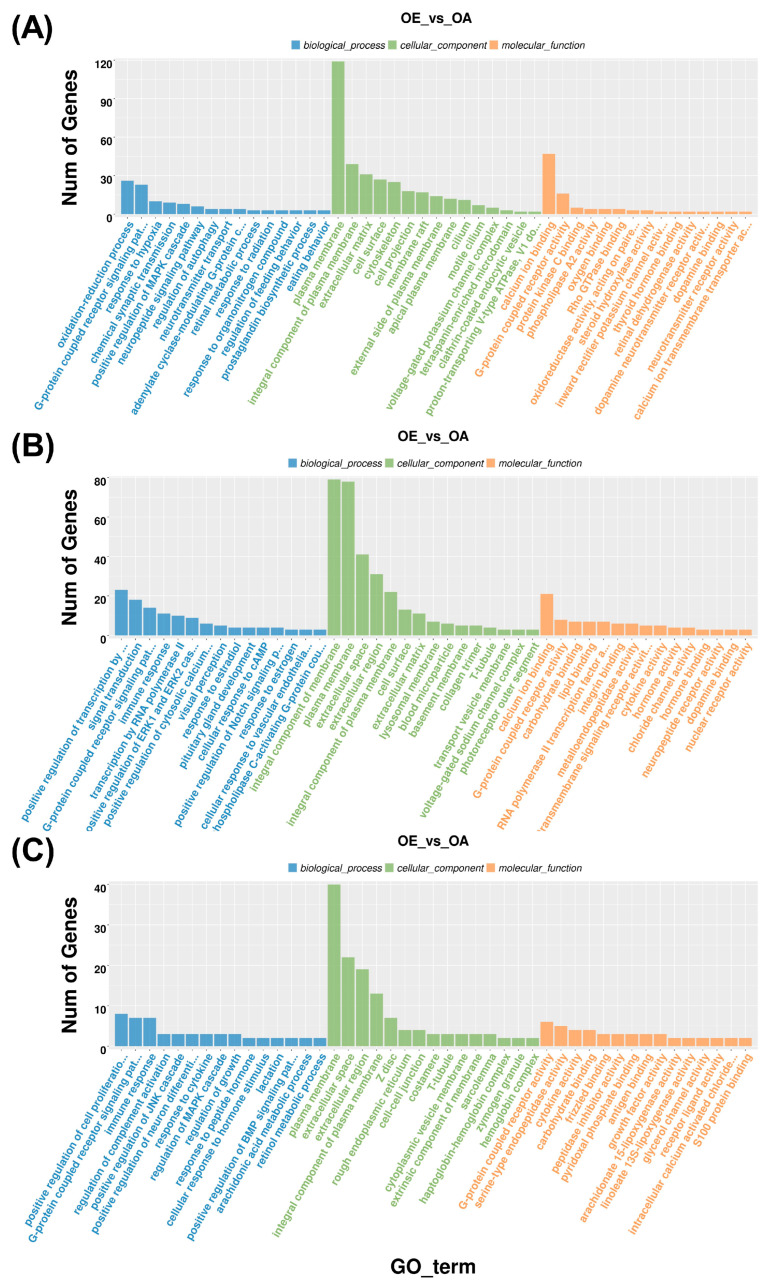
Gene Ontology enrichment analysis of differentially expressed genes in HPO-axis tissues. (**A**) Enriched GO terms in the hypothalamus. (**B**) Enriched GO terms in the pituitary. (**C**) Enriched GO terms in the ovary. Blue represents biological processes, green represents cellular components, and orange represents molecular functions.

To functionally characterize the DEGs, they were annotated within the GO framework, encompassing BP, CC, and MF categories (Figure 2; Supplementary Materials S5). In the hypothalamus, significantly enriched BP terms included the oxidation–reduction process, G-protein-coupled receptor (GPCR) signaling pathway, and response to hypoxia. CC terms were primarily associated with the plasma membrane, extracellular matrix, and cell surface, whereas MF terms involved calcium ion binding and GPCR activity (Figure 2A).

In the pituitary, enriched BP terms were mainly associated with the positive regulation of transcription by RNA polymerase II, signal transduction, and GPCR signaling. CC terms were related to the integral component of membrane, plasma membrane, and extracellular space, while MF enrichment highlighted calcium ion binding, GPCR activity, and carbohydrate binding (Figure 2B).

In the ovary, enriched BP terms included GPCR signaling, regulation of complement activation, and positive regulation of the JNK cascade. CC terms were related to the plasma membrane, extracellular space, and integral component of plasma membrane, while MF terms involved GPCR activity, serine-type endopeptidase activity, and cytokine activity (Figure 2C). Overall, GPCR signaling, plasma membrane localization, calcium ion binding, and GPCR activity were consistently represented across all three HPO-axis tissues.

### 3.4. KEGG Pathway Enrichment of DEGs

**Figure 3:**
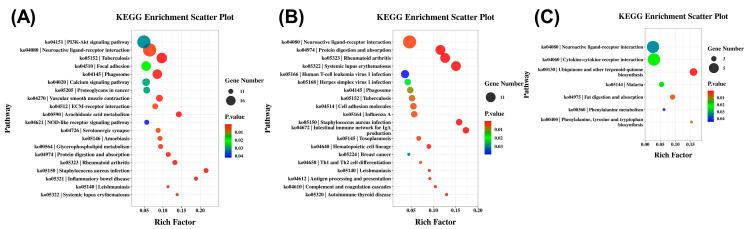
KEGG pathway enrichment analysis of differentially expressed genes in HPO-axis tissues. (**A**) Significantly enriched KEGG pathways in the hypothalamus. (**B**) Significantly enriched KEGG pathways in the pituitary. (**C**) Significantly enriched KEGG pathways in the ovary. Dot size indicates the number of DEGs; color represents the *p*-value range.

KEGG pathway enrichment analysis was performed to identify key signaling pathways involved in seasonal reproduction. In the hypothalamus, the neuroactive ligand–receptor interaction pathway contained the most DEGs, followed by the PI3K–Akt signaling, calcium signaling, and ovarian steroidogenesis pathways (Figure 3A). The pituitary also showed the highest DEG representation in neuroactive ligand–receptor interaction, with additional enrichment in cell adhesion molecules, cytokine–cytokine receptor interaction, and arachidonic acid metabolism (Figure 3B). In the ovary, the neuroactive ligand–receptor interaction and cytokine–cytokine receptor interaction pathways were most prominent, along with reproduction-related pathways such as ubiquinone and other terpenoid–quinone biosynthesis, fat digestion and absorption, and phenylalanine metabolism (Figure 3C). Notably, neuroactive ligand–receptor interaction was the only pathway commonly enriched across all three tissues.

### 3.5. Validation by RT-QPCR

**Figure 4:**
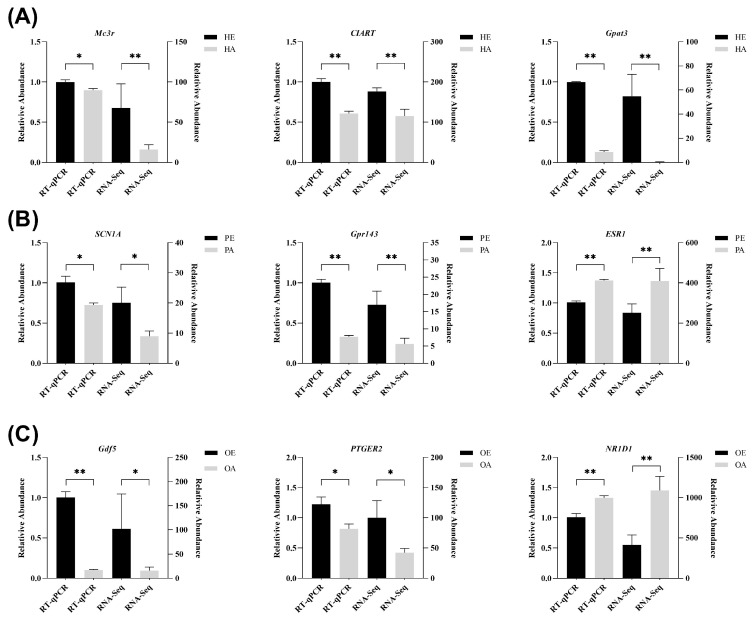
Validation of RNA-seq results by RT-qPCR in HPO-axis tissues. (**A**) Comparison of gene expression levels between RNA-seq and qRT-PCR in the hypothalamus. (**B**) Comparison of gene expression levels between RNA-seq and qRT-PCR in the pituitary. (**C**) Comparison of gene expression levels between RNA-seq and qRT-PCR in the ovary. Significant differences (*p*-value < 0.05) are indicated by one asterisk (*), and highly significant differences (*p*-value < 0.01) are indicated by two asterisks (**).

To verify the RNA-seq results, nine DEGs from the HPO-axis tissues were randomly selected for RT-qPCR validation using *GAPDH* as an internal control. The expression patterns of these genes were consistent with the RNA-seq data (Figure 4), confirming the reliability of the transcriptomic findings for subsequent bioinformatic analyses.

## 4. Discussion

Reproductive function in vertebrates critically depends on the integrated operation of the hypothalamic–pituitary–ovarian (HPO) axis, which synchronizes the development of sexual organs and gametes [16,17]. Because reproductive processes such as folliculogenesis and ovulation are driven by gene expression changes in the hypothalamus, pituitary, and ovaries, RNA transcript profiling provides a powerful approach to elucidate the underlying regulatory mechanisms [18,19]. This study employed RNA-seq technology—widely used for gene discovery and expression analysis in mammalian tissues [20,21]—to identify estrus-related mRNAs across the HPO axis of female Manchurian zokors. Although the absence of a reference genome necessitated a de novo assembly approach, which can introduce issues such as transcript fragmentation and incomplete isoform recovery, these challenges were mitigated through a stringent bioinformatic strategy. This included optimized assembly with Trinity, rigorous multi-database annotation (*E*-value < 0.00001), and RT-qPCR validation of key findings, ensuring the robustness of our dataset [22,23]. This study aimed to establish a molecular basis for understanding how seasonal reproduction is regulated in subterranean rodents living under light-deprived conditions.

As the central hub for environmental signal transduction, differential gene expression in the hypothalamus elucidates how Manchurian zokors translate external environmental cues into reproductive responses. In this study, 513 DEGs were identified in the hypothalamus of female Manchurian zokors during estrus compared with anestrus, comprising 203 upregulated and 310 downregulated genes. GO and KEGG pathway enrichment analyses revealed that the hypothalamus, serving as the integrative center of reproductive rhythm, showed significant enrichment of DEGs in the GO terms neuropeptide signaling pathway (GO:0007218) and adenylate cyclase-modulating G-protein-coupled receptor signaling pathway (GO:0007187). In seasonally breeding sheep, the neuropeptide kisspeptin and its receptor GPR54 are core regulators that activate the mammalian reproductive axis by stimulating GnRH neuronal activity to control ovulation [24,25,26]. This suggests that the Manchurian zokor employs a similar neuroendocrine mechanism to initiate estrus.

The neuroactive ligand–receptor interaction pathway was the only KEGG pathway significantly enriched across all three HPO-axis tissues, highlighting its central role in mediating seasonal estrus in the Manchurian zokor. This pathway has been widely associated with reproductive regulation in vertebrates. Previous transcriptomic studies in ducks, chickens, and fish have demonstrated its involvement in modulating reproductive processes [27,28,29,30,31]. In swine, nutritional restriction has been shown to affect estrus via this pathway [32], while hypothalamic transcriptome analyses in goats have implicated it in sexual maturation [33]. Similarly, a study in Tan sheep reported co-enrichment of this pathway in pineal, hypothalamic, and pituitary tissues, further supporting its conserved function in seasonal reproduction among ruminants [5]. These findings align with our results, reinforcing the notion that the neuroactive ligand–receptor interaction pathway represents an evolutionarily conserved mechanism underlying reproductive timing across diverse seasonal breeders.

Notably, thyrotropin-releasing hormone (*Trh*) and its receptor (*Trhr*) were significantly upregulated during estrus. *Trh*, which plays multifaceted roles in integrating neuroendocrine, autonomic, and reproductive functions, is primarily expressed in the hypothalamic paraventricular nucleus (PVN). Together with its significantly enriched GO terms—response to glucose (GO:0009749) and thyrotropin-releasing hormone activity (GO:0008437)—*Trh* may translate rising temperature cues into accelerated metabolic signals during spring by elevating the thyroid hormone metabolic rate, thereby promoting estrus initiation. Furthermore, as a stimulator of prolactin synthesis, *Trh* may also sustain luteal function via the prolactin pathway or promote ovulation by modulating dopaminergic neuron activity to disinhibit GnRH neurons [34,35,36].

Conversely, the leptin receptor gene (*Lepr*) was significantly downregulated during estrus. Because leptin acts on the hypothalamus to suppress appetite and increase energy expenditure [37], and murine studies show that cold exposure suppresses NPY-5R and activates NPY via upregulation of serum leptin and hypothalamic *Lepr*—thereby downregulating GnRH expression, impairing HPO-axis function, and reducing ovarian reserves [38]—the observed downregulation of *Lepr* may promote estrus by disinhibiting the NPY–GnRH axis. Melanocortin receptor 3 (*Mc3r*) is highly enriched in hypothalamic KNDy neurons in mice [39]. These neurons serve as key hubs regulating GnRH pulse release and, consequently, gonadotropin secretion and the estrous cycle [40]. Studies have reported delayed estrus onset in Mc3r knockout mice. Fasting experiments further confirm that *Mc3r* mediates nutrient-deficiency signals that prolong the estrous cycle, linking energy status to reproduction via leptin signaling [39]. The upregulation of *Mc3r* during estrus in late spring—a period of relatively abundant but not yet peak food availability—may reflect that, although resources improve at estrus onset, substantial vegetation has not yet formed and underground food stores are depleted, leaving Manchurian zokors in relative food scarcity.

Phosphoenolpyruvate carboxykinase 1 (*Pck1*), a rate-limiting enzyme in gluconeogenesis [41], was downregulated during estrus. This downregulation may help prevent hypoglycemia-induced reproductive suppression by reducing the activity of hypothalamic glucose-sensing neurons [42,43]. Pyroglutamylated arginine-phenylalanine-amide peptide receptor (*Qrfpr*), the ligand-binding site of the G-protein-coupled receptor GPR103, plays a central role in regulating energy balance and neuroendocrine activity. The QRFP/QRFPR system significantly promotes feeding, activity, and metabolic rate by activating hypothalamic neurons [44,45]. Because hypothalamic *Qrfp* expression increases during fasting [46], the significant upregulation of *Qrfpr* during estrus suggests that Manchurian zokors integrate energy-status information into estrus-initiation signaling through this receptor.

This study identified 292 DEGs in the pituitary gland of Manchurian zokors, including 115 upregulated and 177 downregulated genes during estrus. Genes associated with pituitary development and reproductive hormone synthesis exhibited significantly higher expression during this phase. Among the upregulated genes, *Pitx2*—a key effector of the Wnt/β-catenin signaling pathway in pituitary development—drives pituitary precursor cell proliferation to establish normal structure and regulates gonadotropin (e.g., LH, FSH) synthesis in the adult mouse pituitary [47,48]. The increased expression of *Pitx2* during estrus may therefore promote gonadotropin release by enhancing gonadotrope cell activity.

Similarly, *NR4A2*—a critical response factor for hypothalamic–pituitary axis activation that is primarily expressed in gonadotropes and induced by GnRH/TRH signaling [49]—was significantly upregulated, suggesting its involvement in regulating gonadotropin synthesis and secretion during estrus. The anterior pituitary differentiation gene *LHX1* was also significantly upregulated during this phase. Given the crucial functions of its homologous genes *LHX3* and *LHX4*—whose deficiency leads to pituitary developmental disorders and impaired gonadotroph formation—*LHX1* may play a similar role during the estrous phase of the Manchurian zokors. *LHX3* can directly bind to the *LHβ*/*FSHβ* promoter to activate transcription, synergize with BMP2 signaling to induce *GATA2* expression, and thereby regulate gonadotropin synthesis [50,51].

Among the downregulated genes during estrus in the pituitary gland, the reduced expression of the core estrogen receptor *ESR1* may promote pulsatile gonadotropin release by attenuating estrogen’s negative feedback [52]. Similarly, the reduced expression of *SIX3* and *SIX6* [53]—regulators of pituitary morphogenesis and inhibitors of the Wnt/β-catenin pathway—may relieve inhibition of the pathway, thereby contributing to estrus initiation. Notably, the *Npy2r* gene, which encodes the neuropeptide Y (NPY) Y2 receptor, was also downregulated. This receptor primarily regulates feeding, energy metabolism, and body-weight control; knockout mice exhibit hyperphagia and excessive weight gain [54,55]. These results underscore the importance of energy-metabolism regulation in the estrous process of Manchurian zokors, consistent with the overall pituitary DEG pattern.

At the ovarian level, 138 DEGs were identified, with 54 upregulated and 84 downregulated during estrus. Among the upregulated genes, prostaglandin E2 receptor subtype 2 (*PTGER2*) showed significantly higher expression. This gene enhances fertilization rates by promoting cumulus expansion during mammalian ovulation; mouse models show that its absence leads to impaired cumulus expansion and a 50% reduction in fertilization rates without affecting uterine receptivity [56,57]. This suggests that *PTGER2* may similarly regulate ovulation in Manchurian zokors.

The sphingosine kinase gene *Sphk1* was also significantly upregulated during estrus in the ovary. Its product, sphingosine-1-phosphate (S1P), is essential for oocyte maturation. LH signaling activates the Sphk1–S1P axis, triggering calcium oscillations and the PI3K/Akt pathway, thereby enhancing oocyte developmental potential [58]. This indicates a potentially positive role for *Sphk1* in oocyte maturation during estrus in Manchurian zokors.

Notably, the cytokine-signaling inhibitors *Socs2* and *Cish* were both upregulated during estrus in the ovary. *Socs2* negatively regulates the GH–JAK2–STAT5b pathway by binding phosphorylated tyrosine residues on the growth hormone receptor (GHR), thereby inhibiting somatic growth [59]. Their upregulation during estrus contrasts with the expectation that the breeding period is typically associated with enhanced metabolism. Combined with DEG patterns in the hypothalamus and pituitary that support estrus onset under low-energy conditions, this suggests that Manchurian zokors may prioritize reproductive processes over somatic growth, adopting a “reproduction-priority” energy allocation strategy.

Among the downregulated genes, the nuclear receptor *NR1D1* (*Rev-erbα*)—a core clock component that inhibits target gene transcription by binding RORE elements—has been shown to negatively regulate estrogen synthesis in porcine ovarian granulosa cells [60]. Its downregulation during estrus may relieve inhibition of estrogen synthesis, providing the hormonal environment necessary for reproductive activity.

Interestingly, despite inhabiting perpetual darkness and possessing a highly degenerate visual system [61], this study detected significant differential expression of several light-responsive genes in the hypothalamic and pituitary transcriptomes of Manchurian zokors. For example, the circadian-associated transcriptional repressor (*Ciart*) was significantly upregulated during estrus. As a core clock repressor directly influenced by light [62], *Ciart* regulates circadian rhythmic output by inhibiting the CLOCK/BMAL1 complex [63].

In the hypothalamus, DEGs were significantly enriched in light-related GO terms such as response to radiation (GO:0009314) and retinal metabolic process (GO:0042574), while in the pituitary, they were enriched in visual perception (GO:0007601) and detection of light stimulus involved in visual perception (GO:0050908). These categories include genes such as *COL2A1*, *Col11a1*, *DLL4*, *EFEMP1*, and *Tulp1*.

Significant upregulation was also observed for *NPAS1*, whose homolog *NPAS2* is a core regulator of mammalian light response and circadian rhythm [64,65,66]; for the light-gated ion channel gene *SCN1A* [67,68]; and for the retinal development-associated gene *GPR143* [69]. These results suggest that although the Manchurian zokor is a strictly subterranean rodent, faint light signals received during brief surface exposure or movement in shallow tunnels may still influence hypothalamic and pituitary function via the SCN. This finding aligns with evidence that the SCN of the Gansu zokor (*Myospalax cansus*) retains photoperiodic perception [61] and that the degenerate eyes of blind subterranean mole-rats (*Spalax*) still express the circadian clock genes Clock and MOP3 (*Bmal1*), indicating residual photic signal perception [70]. Such patterns are consistent with observations in Gansu zokors and blind subterranean mole-rats but contrast with findings in African mole-rats (*Bathyergidae*), which have lost selective pressure on UV damage repair genes due to prolonged subterranean life [71]. While seasonal breeders typically rely on photoperiod to time reproduction, subterranean rodents may have adapted by weakening photoperiodic dependence and strengthening endogenous rhythmicity. This hypothesis is supported by comparative analyses of circadian gene expression between the aboveground *Lasiopodomys brandtii* (*L. brandtii*) and the subterranean *Lasiopodomys mandarinus* (*L. mandarinus*) [72]. The potential role of faint light signals in this regulatory system, though intriguing, remains speculative and requires further validation.

## 5. Conclusions

In this study, RNA-seq was used to identify genes and signaling pathways involved in regulating seasonal reproduction across the HPO axis of the Manchurian zokor. The neuroactive ligand–receptor interaction pathway was significantly enriched in all three HPO-axis tissues, indicating its central role in mediating seasonal estrus. Key candidate genes were identified in each tissue—*Trh* and *Mc3r* in the hypothalamus, *Pitx2* and *NR4A2* in the pituitary, and *PTGER2* and *Sphk1* in the ovary—all closely associated with seasonal reproductive regulation in this subterranean rodent. These findings improve understanding of the molecular regulation mechanisms underlying seasonal reproduction in fossorial mammals and provide a valuable resource for future research on reproductive adaptation in subterranean species. Although core genes and key signaling pathways regulating seasonal reproduction across the HPO axis were identified, the specific molecular mechanisms by which they coordinate estrus initiation in the absence of light cues remain unclear. Functional and mechanistic analyses of these candidate genes and pathways will therefore be essential in future investigations.

## Figures and Tables

**Table 1:** Sample quality control statistics.

Sample	Raw_Reads	Valid_Reads	Valid%	Q20%	Q30%	GC%
HA_1	39,659,208	38,577,930	97.27	98.28	94.91	47.55
HA_2	40,108,460	39,110,952	97.51	98.31	94.91	47.40
HA_3	45,233,794	44,191,340	97.70	98.48	95.34	47.21
HA_4	43,329,156	42,210,708	97.42	98.33	94.97	47.62
HA_5	37,622,004	36,528,498	97.09	98.18	94.54	47.81
HE_1	42,220,284	41,172,088	97.52	98.33	94.96	47.51
HE_2	44,554,920	43,525,226	97.69	98.54	95.52	47.36
HE_3	34,298,706	33,402,070	97.39	98.09	94.39	48.13
HE_4	42,184,640	41,217,140	97.71	98.24	94.72	48.13
HE_5	40,441,964	39,401,858	97.43	98.33	95.05	47.78
OA_1	42,236,070	41,343,484	97.89	98.42	95.22	48.07
OA_2	40,477,686	39,551,868	97.71	98.37	95.15	48.84
OA_3	44,373,928	43,408,348	97.82	98.47	95.38	49.11
OA_4	41,722,314	40,773,392	97.73	98.40	95.15	48.35
OA_5	43,050,446	41,998,778	97.56	98.47	95.40	47.86
OE_1	46,817,224	45,654,450	97.52	98.48	95.42	48.97
OE_2	44,016,598	43,024,650	97.75	98.42	95.23	48.36
OE_3	41,430,944	40,403,572	97.52	98.36	95.09	48.43
OE_4	42,306,882	41,298,450	97.62	98.33	94.98	48.20
OE_5	40,895,868	39,921,076	97.62	98.35	95.03	48.35
PA_1	40,961,408	39,822,102	97.22	98.13	94.33	47.12
PA_2	40,893,564	39,866,926	97.49	98.35	94.93	47.96
PA_3	34,333,216	33,316,172	97.04	98.13	94.36	47.52
PA_4	41,997,552	40,841,310	97.25	98.34	94.96	47.41
PA_5	40,709,220	39,739,488	97.62	98.35	95.02	47.26
PE_1	41,099,134	40,133,734	97.65	98.38	95.16	47.88
PE_2	41,343,140	40,378,170	97.67	98.32	94.95	47.68
PE_3	41,326,142	40,262,252	97.43	98.36	95.13	47.30
PE_4	42,633,884	41,645,678	97.68	98.34	94.93	47.73

## Data Availability

The original contributions presented in this study are included in the article/Supplementary Material. Further inquiries can be directed to the corresponding authors.

## Supplementary Materials

The following supporting information can be downloaded at: https://www.mdpi.com/article/10.3390/genes16111289/s1, Supplementary Materials S1: real-time fluorescence quantification primer sequence. Supplementary Materials S2: HE_vs_HA_Differentially Expressed Genes. Supplementary Materials S3: PE_vs_PA_Differentially Expressed Genes. Supplementary Materials S4: OE_vs_OA_Differentially Expressed Genes. Supplementary Materials S5: GO entries for differentially expressed genes. Supplementary Materials S6: KEGG pathway entries for differentially expressed genes.

## Abbreviations

BP	biological processes
CC	cellular components
Ciart	circadian-associated transcriptional repressor
DEGs	differentially expressed genes
FSH	follicle-stimulating hormone
GO	Gene Ontology
GnRH	gonadotropin-releasing hormone
GPCR	G-protein-coupled receptor
GHR	growth hormone receptor
HPO	hypothalamic–pituitary–ovarian
KEGG	Kyoto Encyclopedia of Genes and Genomes
LH	luteinizing hormone
Mc3r	Melanocortin receptor 3
MF	molecular functions
NPY	neuropeptide Y
PVN	paraventricular nucleus
Pck1	Phosphoenolpyruvate carboxykinase 1
PTGER2	prostaglandin E2 receptor subtype 2
Qrfpr	Pyroglutamylated arginine-phenylalanine-amide peptide receptor
qRT-PCR	quantitative real-time PCR
S1P	sphingosine-1-phosphate
Trh	thyrotropin-releasing hormone
