# Supplementary material for: Molecular Characterization of Hypothalamic–Pituitary–Ovarian Axis Regulation in the Manchurian Zokor (Myospalax psilurus) During Seasonal Estrus
Source: Genes (Basel). 2025 Oct 30;16(11):1289. doi: 10.3390/genes16111289 (PMC12652709; doi:10.3390/genes16111289)
Supplement: Supplementary file 1 [file genes-16-01289-s001.zip › S1 real-time fluorescence quantification primer sequence.pdf]

Table S1. Hypothalamic real-time fluorescence quantitative primer sequence

mRNA		Sequence（5' - 3'）	Temperature(℃)
Mc3r	F	TTCTGTCATCGACCCCCTCA	60.25
	R	CAGCCCTAGCCCAAGTTCAT	59.74
CIART	F	GGCAGGATCTGGGGTCAAAA	59.96
	R	TTTTGCGCAAAGAGCAGGTC	59.97
Gpat3	F	GGAACCTCCTCACACGAACC	60.32
	R	CCAGTTTTTGAGGCTGCTGTC	60.00

Table S2. Pituitary real-time fluorescence quantitative primer sequence

mRNA		Sequence（5' - 3'）	Temperature(℃)
ESR1	F	GCTTTGGGGACTTAAACCTGC	59.73
	R	TTAAGGGTGCTGTCCAAGAGCAA	62.34
SCN1A	F	TTGACCTGGCCATCACCATC	60.03
	R	TCCTTCCACATTGGCGAGTC	60.04
Gpr143	F	TCCCTGAGCAAGACGAATGG	59.75
	R	CTGATAACGATTCCCAGGCAG	58.51

Table S3. Ovarian real-time fluorescence quantitative primer sequence

mRNA		Sequence（5' - 3'）	Temperature(℃)
NR1D1	F	TTGGAGACTTCCCGCTTCAC	59.97
	R	GCAGCTTCTCGGAATGCATG	59.97
Gdf5	F	GAGATTAAGGCCCGCTCTGG	59.96
	R	CTCACACAGTCCTTCGCAGT	59.89
PTGER2	F	CTGCTGCTGCTTCTCATCGT	60.47
	R	ACTTACTCCGCAGGCATCTG	59.82
